# Current Knowledge, Attitudes, and Practices of Home Health Care Among Primary Health Care Physicians in Al-Ahsa, Saudi Arabia: A Cross-Sectional Study

**DOI:** 10.7759/cureus.91598

**Published:** 2025-09-04

**Authors:** Asma AlDrees, Sawsan AlSalem, Ola AlDrees, Ahmed AlHaddad

**Affiliations:** 1 Family Medicine, Al-Ahsa Family Medicine Academy, Al-Ahsa, SAU; 2 Family Medicine, Al-Ahsa Health Cluster, Ministry of Health, Al-Ahsa, SAU

**Keywords:** family medicine, home care, home care patient, home health care, primary health care physicians

## Abstract

Introduction

Home health care (HHC) is designed to assist individuals in improving their function and live with greater independence, promote optimal health, help patients remain at home, avoid hospitalization, and prolong expensive hospital care. Primary health care (PHC) physicians play an essential role in providing continuity of care for HHC patients. However, there is a deficiency in medical education and training.

Aim

This study aimed to document the knowledge, attitudes, and practices of HHC among PHC physicians in Al-Ahsa, Saudi Arabia.

Methods

This descriptive cross-sectional questionnaire-based study was conducted in the Al-Ahsa region of Saudi Arabia between February and July 2025. The target population included all PHC physicians (family medicine consultants, specialists, residents, and general practitioners) working at the Ministry of Health (MOH) PHC centers in Al-Ahsa. The study involved 220 PHC physicians. Participants were selected using simple random sampling, and a self-administered questionnaire was used as an instrument for data collection. Data were analyzed using the Statistical Package for the Social Sciences (SPSS) (IBM SPSS Statistics for Windows, IBM Corp., Version 27, Armonk, NY). Both descriptive and inferential statistics were used, with a p-value ≤ 0.05 considered significant in all tests.

Results

Of the 220 participants, most were female (63.6%), aged 20-30 years (56.8%), and family medicine residents (39.5%). Only 10% of the participants had formal HHC training. Knowledge was generally poor, with only 3.6% of respondents demonstrating good knowledge. Attitudes were modest, with 20.9% showing a positive attitude toward HHC. Only 15.5% of the participants had a good practice score. The most reported barriers were lack of experience (34.1%) and unavailable service (18.2%). There was a significant association between formal training and greater years of experience were significantly associated with higher knowledge, attitude, and practice (KAP) scores (p < 0.001).

Conclusion

The results of this study indicate low KAP scores among PHC physicians in Al Ahsa, Saudi Arabia. The findings of this study highlight the need for systemic improvements in HHC training and integration within PHC settings. Exposing PHC physicians to the HHC field will empower them to deliver comprehensive, coordinated, and high-quality home-based care services.

## Introduction

As the population ages, there is more demand for health services and hospital costs [1]. Home health care (HHC) is defined as “a system of care provided by skilled practitioners to patients in their homes under the direction of a physician” [2].

The concept of HHC was first generated to provide postpartum care and care for patients with infectious diseases in the 19th century. Then, in the mid-20th century, it changed to include caring for elderly patients [3]. In the Kingdom of Saudi Arabia, in 1991, the first HHC program was established for patients with advanced cancer at King Faisal Specialized Hospital and Research Center. Another HHC program was established in 2008 by the Saudi MOH [4]. This program provides therapeutic, preventive, educational, rehabilitation, and social services by a qualified medical team under the regulation of global standards and Islamic values [5].

According to the Saudi General Authority for Statistics, the elderly, people aged 65 years and older, make up about 4.2% of the population. In addition, 74.1% of the elderly have a chronic disease, and 79.4% use health services provided by the government [6]. Thus, the need for home care services arises. Although HHC is primarily used in the older population, it is available for people of all ages who require preventive, acute, subacute, rehabilitative, and long-term care [7].

HHC offers home-based care for a range of conditions, including wound care for bedsores, chronic disease management, and palliative care. Physicians at HHC also perform medical procedures such as parenteral nutrition, Foley catheter changes, and medication injections [8]. HHC aims to help individuals improve their function, live more independently, and maintain optimal health at home. This care is designed to prevent hospitalizations and reduce the need for costly, prolonged hospital stays or institutional care [9].

Eligibility for HHC services includes patients who are discharged from the hospital with no full recovery, patients with chronic conditions, terminal illness, limited mobility, and requiring special life-sustaining equipment [8]. Qualitative research was done in Norway from June 2015 to June 2016 by Danielsen et al. [10], aiming to study the challenges faced by home palliative care nurses and general practitioners. It used a semi-structured question guide interview with four focus groups with 19 participants. This study showed that effective collaboration between patients, families, and health practitioners reduced readmission rates. Nevertheless, it emphasizes the need for safer discharge protocols with good timing for transferring the patient to home [10].

A cross-sectional study conducted by Alkeridy et al. [11] among Saudi people receiving long-term HHC services at King Saud University Medical City (KSUMC), Riyadh, Saudi Arabia, showed that the mean age of deceased individuals in HHC was 78.3 years. Over 20% of individuals receiving HHC services were readmitted to the hospital. They found that the strongest predictor for mortality was pressure ulcers with an odds ratio of 3.75 and a p-value of <0.0001. In conclusion, the study found that pressure ulcers and frailty are the strongest predictors of mortality among individuals receiving HHC services [11].

Primary health care (PHC) physicians play an essential part in providing continuity of care for HHC patients [12]. However, there is a deficiency in medical education and training [13]. HHC requires proper training and supervision in order to provide a safe environment for the patients and healthcare staff, which is necessary for the success of HHC services. Untrained healthcare physicians who lack expertise cannot deliver the services needed, affecting patient safety [14]. Receiving HHC services can be made either by a physician’s referral or by the family/patient’s request [8].

Limited studies were done in Saudi Arabia that assessed the knowledge, training, and attitudes of PHC physicians toward HHC. Therefore, it is crucial to explore the practice of PHC physicians toward HHC in Saudi Arabia. This study aims to document the knowledge, attitudes, and practices of HHC among PHC physicians in Al Ahsa, Saudi Arabia.

## Materials and methods

Study design and setting

This descriptive cross-sectional questionnaire-based study was conducted in the Al-Ahsa region of Saudi Arabia between February and July 2025.

Study population

The target population included all PHC physicians (family medicine consultants, specialists, residents, and general practitioners) working at the Ministry of Health (MOH) PHC centers in Al-Ahsa. Physicians who were on vacation or who had participated in the pilot study were excluded. 

Sampling technique

The sample size was calculated using Raosoft software (Raosoft Inc., Seattle, WA), with a 95% confidence level, 5% margin of error, and an assumed prevalence of 50%, based on a total estimated physician population of 500 in Al-Ahsa in 2024. The final sample size was 220 physicians, selected using a simple random technique from the sample frame.

Data collection

A self-administered questionnaire (Appendices), validated by experts and pre-tested in a pilot study, was used to collect the data that aimed to assess knowledge, attitude, and practice (KAP) regarding HHC among PHC physicians. The questionnaire consisted of four sections: socio-demographic data (including gender, education, years of experience, prior HHC training, exposure to HHC lectures, and work in HHC departments); 18 items assessing knowledge; five items assessing attitude; and three items assessing practice. Internal consistency was confirmed using Cronbach’s alpha (α = 0.87), and content validity was reviewed by four experts in family medicine.

Data analysis

Data were analyzed using SPSS (IBM SPSS Statistics for Windows, IBM Corp., Version 27, Armonk, NY) [15]. Descriptive statistics were used to present means with standard deviations for continuous variables and frequencies with percentages for categorical variables. Normality testing indicated that the data were not normally distributed; hence, mean rank was calculated by the Mann-Whitney U test, which was used for comparing KAP scores with dichotomous categorical variables, and the Kruskal-Wallis test, which was used for variables with more than two categories. A p-value ≤ 0.05 was considered statistically significant. KAP scores were categorized using Bloom’s cut-off points as low (<60%), moderate (60-79%), or good (≥80%). Ethical approval was obtained from the institutional review board. Participation was voluntary, and informed consent was obtained electronically before accessing the questionnaire. All participant data were kept confidential and used solely for research purposes.

## Results

Table 1 shows that the study involved 220 healthcare professionals to assess their knowledge, attitudes, and practices regarding HHC. More than half of them (51.8%) were female, more than half (56.8%) were aged 20-30 years, more than one-third (39.5%) were residents, and 29.1% were specialists. More than two-thirds (67.3%) have less than five years of experience. Most of the participants (90.0%) reported not having training in HHC, 75.9% not attending HHC lectures, and 90.9% have no prior work experience in HHC departments.

**Table 1:** Sociodemographic characteristics

Sociodemographic characteristics		Frequency (percentage)
Gender	Male	106 (48.2%)
	Female	114 (51.8%)
Age	20-30	125 (56.8%)
	31-40	83 (37.7%)
	More than 40	12 (5.5%)
Occupation	GP	33 (15.0%)
	Resident	87 (39.5%)
	Specialist	64 (29.1%)
	Consultant	36 (16.4%)
Years of experience	Less than five years	148 (67.3%)
	Five to 10 years	43 (19.5%)
	More than 10 years	29 (13.2%)
Training	Yes	22 (10.0%)
	No	198 (90.0%)
I have attended lectures about home health care	Yes	53 (24.1%)
	No	167 (75.9%)
Did you work in home health care department before?	Yes	20 (9.1%)
	No	200 (90.9%)

Table 2 shows that only 20.0% of participants rated that they had enough knowledge about HHC; there was a strong understanding that HHC services are not limited to the elderly (76.8%). Furthermore, a majority of participants correctly identified that caring for patients with chronic diseases is one of the HHC services (86.8%). Similarly, caring for patients with pressure ulcers was recognized as an HHC service by 87.7% of respondents. Participants also showed high awareness that HHC services include providing medicines, medical supplies, and supplements for patients (90.5%). Additionally, 78.2% correctly stated that HHC provides palliative care. While general knowledge of HHC was mixed, participants demonstrated strong awareness of several specific medical services. A majority of respondents (80.5%) knew that HHC cares for urinary catheterization patients, and 77.7% recognized that HHC cares for enteral nutrition patients (those on nasogastric tube (NGT) and percutaneous endoscopic gastrostomy (PEG) feeding). Knowledge of HHC providing laboratory tests was also high (78.2%). Moreover, 70.5% of participants correctly identified that HHC provides physiotherapy services, and 65.9% were aware that HHC provides intravenous therapy services. A majority (71.8%) also correctly stated that HHC provides psychological and social support. However, knowledge regarding other specific HHC services was notably lower. Only 23.6% correctly knew that HHC provides postpartum care. Similarly, only 38.6% recognized that HHC provides oral health services. Knowledge about HHC arranging non-emergency transportation for non-ambulatory patients was moderate, with 63.2% aware, but a considerable 27.7% were unsure. Less than half of the participants (47.3%) correctly knew that pressure ulcers are staged into three stages, while 63.2% correctly identified that the FRAIL scale is used to assess risk for falls and dizziness in the elderly [16]. Conversely, there was widespread misinformation regarding the STEADI questionnaire, with only 35.0% correctly answering "No" to the statement that it is used to assess depression in the elderly [17].

**Table 2:** Knowledge items about home health care HHC: home health care; NGT: nasogastric tube; PEG: percutaneous endoscopic gastrostomy

Knowledge items		Yes	No	I don't know
I have enough knowledge about HHC.	N (%)	44 (20.0%)	131 (59.5%)	45 (20.5%)
Home health care services are limited only to the elderly.	N (%)	27 (12.3%)	169 (76.8%)	24 (10.9%)
One of the HHC services is to care for patients with chronic diseases.	N (%)	191 (86.8%)	18 (8.2%)	11 (5.0%)
What are the services applied in HHC? (choose from below)/who is eligible to get benefit from HHC services?				
One of the HHC services is to care for patients with pressure ulcers.	N (%)	193 (87.7%)	10 (4.5%)	17 (7.7%)
One of HHC services is providing medicines, medical supplies and supplements for patients.	N (%)	199 (90.5%)	11 (5.0%)	10 (4.5%)
HHC provides palliative care.	N (%)	172 (78.2%)	20 (9.1%)	28 (12.7%)
HHC provides postpartum care.	N (%)	52 (23.6%)	98 (44.5%)	70 (31.8%)
HHC provides oral health.	N (%)	85 (38.6%)	59 (26.8%)	76 (34.5%)
HHC provides intravenous therapy services.	N (%)	145 (65.9%)	31 (14.1%)	44 (20.0%)
HHC provides physiotherapy services.	N (%)	155 (70.5%)	23 (10.5%)	42 (19.1%)
HHC cares for urinary catheterization patients.	N (%)	177 (80.5%)	15 (6.8%)	28 (12.7%)
HHC cares for enteral nutrition patients (patients on NGT and PEG feeding).	N (%)	171 (77.7%)	12 (5.5%)	37 (16.8%)
HHC provides laboratory tests.	N (%)	172 (78.2%)	16 (7.3%)	32 (14.5%)
HHC provides psychological and social support.	N (%)	158 (71.8%)	18 (8.2%)	44 (20.0%)
HHC arranges non-emergency transportation for non-ambulatory patients.	N (%)	139 (63.2%)	20 (9.1%)	61 (27.7%)
Pressure ulcers are staged into three stages.	N (%)	104 (47.3%)	49 (22.3%)	67 (30.5%)
FRAIL scale is used to assess risk for fall and dizziness in elderly.	N (%)	139 (63.2%)	19 (8.6%)	62 (28.2%)
The STEADI questionnaire is used to assess depression in elderly.	N (%)	77 (35.0%)	45 (20.5%)	98 (44.5%)

Table 3 reveals that more than half (63.2%) either agree (40.9%) or strongly agree (22.3%) that they have a clear understanding of their role in HHC. While 30.9% agreed they had confidence in completing referral requests. However, a notable proportion also reported being "uncertain" (25.0%). About one-third (32.7%) of participants agreed that they are confident in evaluating wounds and managing them accordingly, and 31.4% were confident in performing a complete mental status examination. Fewer than a quarter of participants (24.1%) reported having the confidence to perform a complete, comprehensive geriatric assessment.

**Table 3:** Attitude items about home health care

Attitude items		Strongly disagree	Disagree	Uncertain	Agree	Strongly agree
I have a clear idea of the role of home health care in my specialty.	N (%)	27 (12.3%)	41 (18.6%)	90 (40.9%)	49 (22.3%)	13 (5.9%)
I am confident to complete referral request to home health care for patients.	N (%)	28 (12.7%)	38 (17.3%)	55 (25.0%)	68 (30.9%)	31 (14.1%)
I am confident to evaluate wound and manage them accordingly.	N (%)	30 (13.6%)	42 (19.1%)	61 (27.7%)	72 (32.7%)	15 (6.8%)
I am confident to do complete mental status examination.	N (%)	25 (11.4%)	32 (14.5%)	65 (29.5%)	69 (31.4%)	29 (13.2%)
I am confident to do complete comprehensive geriatric assessment.	N (%)	28 (12.7%)	39 (17.7%)	69 (31.4%)	53 (24.1%)	31 (14.1%)

Table 4 shows the practice of HHC, regarding the frequency of referring patients to HHC, the most common responses were sometimes (30.9%) and never (29.1%). Concerning direct involvement in providing care to HHC patients at their home, the majority of participants (64.1%) reported never engaging in this practice. Before referring patients to HHC, a strong majority of participants consistently perform pre-referral assessments. Taking a detailed history was most frequently reported as always (55.9%), physical examination (46.4%), and writing a detailed plan of care before referral (41.8%). More than one-fifth reported ease of communicating with providers of HHC, always. Concerning the ability to adjust the plan of care for patients under HHC, the most common response was never (29.1%), followed closely by always (20.5%) and often (20.0%).

**Table 4:** Practice items regarding home health care HHC: home health care

Practice items		Never	Seldom	Sometimes	Often	Always
I have referred patients to HHC.	N (%)	64 (29.1%)	30 (13.6%)	68 (30.9%)	34 (15.5%)	24 (10.9%)
I have provided care to home health care patients at their home.	N (%)	141 (64.1%)	21 (9.5%)	21 (9.5%)	17 (7.7%)	20 (9.1%)
I take detailed history before referring patients to home health care.	N (%)	31 (14.1%)	8 (3.6%)	19 (8.6%)	39 (17.7%)	123 (55.9%)
I perform physical examination before referring patients to home health care.	N (%)	31 (14.1%)	11 (5.0%)	36 (16.4%)	40 (18.2%)	102 (46.4%)
I write detailed plan of care before referring patients to home health care.	N (%)	35 (15.9%)	15 (6.8%)	27 (12.3%)	51 (23.2%)	92 (41.8%)
I can easily communicate with providers of home health care.	N (%)	72 (32.7%)	17 (7.7%)	40 (18.2%)	45 (20.5%)	46 (20.9%)
I can adjust on the plan of care of patients under home health care.	N (%)	64 (29.1%)	29 (13.2%)	38 (17.3%)	44 (20.0%)	45 (20.5%)
How often do you refer patients to HHC per month.	N (%)	64 (29.1%)	76 (34.5%)	71 (32.3%)	7 (3.2%)	2 (0.9%)

Table 5 shows that about one-half of the participants reported there were barriers to practicing HHC. The most prominent barrier identified is a lack of experience in HHC (34.1%), followed by the unavailability of services (18.2%). Regarding sources of information about HHC, the most commonly mentioned sources are personal or professional experience (81.8%), self-study (47.3%). Conversely, formal channels like workshops/lectures (26.8%) and especially the media (11.8%) play a much smaller role in informing individuals about HHC.

**Table 5:** Barriers and sources of information regarding home health care *Multiple answers HHC: home health care

Variables		Frequency (percent)
Is there barrier for HHC?	Yes	106 (48.2%)
	No	114 (51.8%)
Mention barriers*	Number of patients	32 (14.5%)
	Service not available	40 (18.2%)
	No experience in HHC	75 (34.1%)
Source of information regarding HHC*	Media	26 (11.8%)
	Workshops/lecture	59 (26.8%)
	Experience	180 (81.8%)
	Self-study	104 (47.3%)

Table 6 shows that more than half of the participants (52.7%) demonstrated poor knowledge, 43.6% exhibited a moderate level of knowledge, while only 3.6% displayed good knowledge. Regarding attitudes, less than half of participants (43.6%) held a neutral attitude, followed by 35.5% expressed a negative attitude, while 20.9% held a positive attitude. In terms of practice, less than half (42.7%) demonstrated poor practice, 41.8% exhibited a moderate, and 15.5% of participants showed good practice.

**Table 6:** Knowledge, attitude, and practice scores

Items		Frequency (percent)
Knowledge	Poor	116 (52.7%)
	Moderate	96 (43.6%)
	Good	8 (3.6%)
Attitude	Negative	78 (35.5%)
	Neutral	96 (43.6%)
	Positive	46 (20.9%)
Practice	Poor	94 (42.7%)
	Moderate	92 (41.8%)
	Good	34 (15.5%)

Table 7 explores how various demographic and professional characteristics are associated with participants' KAP scores.

**Table 7:** Relationship between knowledge, attitude, and practice domains and sociodemographic characteristics *Statistically significant

Variables		N	Knowledge		Attitude		Practice	
			Mean rank	p-value	Mean rank	p-value	Mean rank	p-value
Gender	Male	106	123.2	0.004*	118.5	0.070	115.2	0.294
	Female	114	98.7		103.0		106.2	
Age	20-30	125	101.6	0.022*	100.0	0.011*	102.4	0.005*
	31-40	83	118.9		127.0		115.2	
	More than 40	12	145.8		106.2		162.8	
Job	GP	33	101.2	<0.001	92.9	0.010*	106.5	0.333
	Resident	87	89.6		99.4		102.3	
	Specialist	64	124.3		124.0		117.9	
	Consultant	36	144.9		129.5		120.9	
Experience	Less than five years	148	97.7	<0.001	102.7	0.029*	106.8	0.123
	Five to 10 years	43	140.2		129.4		108.2	
	More than 10 years	29	131.8		122.5		133.0	
Training	Yes	22	141.8	0.015*	123.9	0.298	164.6	<0.001
	No	198	107.0		109.0		104.5	
Attending	Yes	53	146.6	<0.001	127.7	0.024*	154.5	<0.001
	No	167	99.0		105.1		96.5	
Work	Yes	20	124.0	0.318	127.5	0.210	165.3	<0.001
	No	200	109.2		108.8		105.0	

Gender played a significant role in knowledge scores (p = 0.004). Male participants had a higher mean rank (123.2) compared to females (98.7). However, gender did not significantly influence attitude (p = 0.070) or practice (p = 0.294) scores.

Age was significantly associated with knowledge (p = 0.022), attitude (p = 0.011), and practice (p = 0.005) scores. The "more than 40" age group consistently showed the highest mean ranks across all three domains. Job role had a significant impact on both knowledge (p < 0.001) and attitude (p = 0.010) scores. Consultants exhibited the highest mean ranks for knowledge (144.9) and attitude (129.5). Conversely, residents had the lowest mean ranks for knowledge (89.6) and GPs for attitude (92.9). However, job role did not significantly affect practice scores (p = 0.333).

Years of experience showed a significant association with knowledge (p < 0.001) and attitude (p = 0.029) scores. Participants with five to 10 years of experience had the highest mean rank for knowledge (140.2) and had a higher mean rank for attitude (129.4). While less than five-year group consistently showed the lowest mean ranks for knowledge (97.7) and attitude (102.7). There was no significant association between experience and practice scores (p = 0.123).

Having received training was significantly associated with knowledge (p = 0.015) and practice (p < 0.001) scores. Participants who received training had considerably higher mean ranks for both knowledge (141.8) and practice (164.6) compared to those who did not. However, training did not significantly influence attitude scores (p = 0.298).

Attending relevant events (e.g., conferences, workshops) was significantly associated with knowledge (p < 0.001), attitude (p = 0.024), and practice (p < 0.001) scores. Participants who attended consistently showed much higher mean ranks across all three domains (knowledge: 146.6; attitude: 127.7; practice: 154.5) compared to those who did not attend.

Finally, working in HHC did not significantly influence knowledge (p = 0.318) or attitude (p = 0.210) scores. However, it was significantly associated with practice scores (p < 0.001), with those working in HHC showing a higher mean rank for practice (165.3) compared to those who did not (105.0).

## Discussion

This was a descriptive cross-sectional study to assess PHC physicians’ knowledge, attitudes, and practice of HHC in Al-Ahsa, Eastern Saudi Arabia. Most participants were in the reproductive age group, with more than half of them (56.8%) falling between the ages of 20 and 30. The participants were mostly family medicine residents (39.5%), followed by specialists (29.1%), consultants (16.4%), and general practitioners (15.0%). Most of the participants (67.3%) had less than five years of experience.

The current study revealed that the majority of our participants (90.0%) had not received any official training in HHC, most of them (75.9%) had never attended HHC-related lectures, and most of them (90.9%) reported no previous work experience in HHC departments. Our findings were consistent with earlier research conducted in Saudi Arabia by Al-Hazmi et al. [18]. It showed that most Saudi healthcare professionals had minimal training, and their knowledge was gained through hospital experience rather than accredited education. This general lack of experience and training in HHC among participants suggests a systemic issue in professional preparation and development, particularly for PHC professionals expected to provide such care. Raetz et al. similarly reported that family medicine residents in the United States are often unaware of their role in HHC due to insufficient curriculum integration and lack of clinical exposure [12].

Our study reveals a significant gap in the general knowledge of PHC professionals regarding HHC services. The majority of the participants showed poor knowledge regarding HHC (52.7%), and only 3.6% showed good knowledge. A study done by Choe et al. to assess knowledge, attitude toward hospital-at-home (HaH) showed 51% of participants had good knowledge of HaH [19]. Only 20% of the participants reported that they had sufficient knowledge. However, many participants showed good awareness of certain specific HHC services. This suggests that while practitioners may be knowledgeable about certain clinical aspects of HHC, they lack a comprehensive and integrated understanding of the full scope and objectives of home-based care delivery. Similarly, a previous study by Al-Hazmi et al. showed little knowledge of HHC services among health professionals [18]. Additionally, high recognition of services like urinary catheterization (80.5%), enteral nutrition (77.7%), and laboratory testing (78.2%) reflects familiarity with common clinical tasks, which are likely integrated into their hospital-based roles. However, lower awareness levels regarding postpartum care (23.6%) and oral health services (38.6%) point to persistent knowledge gaps about HHC’s multi-disciplinary and preventive care dimensions. These gaps may be due to the absence of formal education and training of HHC in the undergraduate and residency curriculum, as observed by Raetz et al., who stressed the importance of embedding home care principles into family medicine training [12]. Furthermore, only one third (35.0%) of participants in our study correctly answered the purpose of geriatric assessment, such as the STEADI questionnaire, which highlights the lack of familiarity with specialized assessment tools for community-based elderly care. A study done in Saudi Arabia by Alkeridy et al. showed that pressure ulcers and frailty are the strongest predictors of mortality for individuals receiving HHC services [11]. Our study shows a relatively strong awareness of palliative care (78.2%) and psychosocial support (71.8%), showing growing recognition of HHC’s role in enhancing quality of life rather than providing clinical interventions. Overall, the limited general knowledge and partial understanding of specific services support the urgent need for formal HHC education and structured professional development. As emphasized by Al-Hazmi et al., successful implementation of HHC services in Saudi Arabia requires that providers not only understand the technical aspects of care but also embrace a holistic, patient-centered model [18].

In our study, there was a significant correlation between the knowledge score and the gender of the participants, in which male participants showed a higher mean knowledge score than female participants. Furthermore, age, job title, and years of experience significantly influenced knowledge scores. For example, consultants and physicians with more than five years of experience had higher knowledge and attitude scores. Our findings are consistent with a cross-sectional study conducted in northwest Ethiopia to assess the knowledge of nurses working in adult critical care units regarding geriatric care. Their findings indicated that the level of education and years of experience were significantly associated with higher knowledge of geriatric care [20]. A study conducted in Saudi Arabia [21] and Addis Ababa, Ethiopia [22] demonstrated that the level of education and years of experience were significantly associated with higher knowledge of geriatric care.

As for attitude, only 20% of the participants demonstrated a positive attitude toward various aspects of HHC. This raises concerns, as poor attitudes may reflect limited exposure to HHC services. Participants aged 31-40 years showed significantly better attitudes toward HHC. The study indicates noticeable uncertainty among healthcare professionals regarding their roles and responsibilities in HHC. This limited confidence is supported by the findings of other studies in similar settings [18]. Our findings partially align with those of a recent study conducted in Singapore among internal medicine, family medicine, and emergency medicine residents, which assessed attitudes toward HaH services - a model closely related to HHC. In that study, the overall attitude was neutral [19]. Engaging physicians in HHC training could improve their confidence in identifying and referring patients to HHC services. Furthermore, similar to the knowledge score, age, job title, and years of experience significantly influenced the attitude scores. These results are in line with other international reports that demonstrate a positive association between age and higher educational level with attitudes toward geriatric patients [20,23].

Considering practice, our study showed that a minority of our respondents had good HHC practices (15.5%). Our findings are consistent with several studies, such as a study in Nigeria that has reported the moderate practice of care by healthcare providers toward geriatric patients [24]. In addition, a cross-sectional study was conducted to assess the knowledge, attitudes, and practices of medical doctors in Uganda. It was found that most participants failed to follow the recommended management practices for geriatric patients [25]. Furthermore, most participants had referred patients for HHC. Moreover, more than half of the participants had not been involved in the direct care of patients at home. This is in contrast to a study conducted in the United States among family medicine program directors to address HHC training curriculum. The majority of family medicine residents completed two to five HHC visits by the time of graduation [26]. Nevertheless, more than one-fifth of the participants reported ease of communication with HHC providers. In addition, regarding the ability to adjust the health care plan of patients of HHC, the most reported response was never. This indicates poor communication between PHC and HHC providers. Our findings are similar to those of a study conducted in the United States that aimed to determine the communication between HHC providers and physicians. It was found that nearly 40% of HHC providers were never or rarely able to contact physicians [27]. In addition, our study demonstrated that attending workshops, working in HHC, and prior training were associated with better practice scores, highlighting the importance of exposing PHC physicians to the HHC field. This is in line with Orit et al., who demonstrated that training in older people care is positively impacted on medical doctors toward geriatric care [25]. However, experience in HHC departments did not significantly impact knowledge or attitude, but did influence practice scores. This indicates that exposure alone may not be sufficient to improve knowledge unless it is supported by structured training and clear role definitions.

The current study identified that approximately half of the participants (48.2%) reported barriers to HHC practice. The most frequently cited barrier was a lack of experience, followed by the unavailability of services. These findings are similar to those reported in prior studies in which it was found that inadequate geriatric knowledge and a lack of hospital guidelines on geriatric care are associated with suboptimal treatment practice [25,28-30]. Moreover, a qualitative study conducted by Zhang et al. [31] in China explored barriers to providing home-based hospice and palliative care among community nurses. It was highlighted that a lack of clinical training and experience was a major obstacle to effective home-based care. This consistency highlights a broader systemic issue: the underrepresentation of home care training in clinical education programs across different healthcare systems. Additionally, both studies pointed to resource limitations, including the unavailability of home health services, as major impediments [31]. Furthermore, a study conducted in the United States among family medicine program directors found that the greatest perceived barrier for residents to train in HHC was a lack of interest and the complex logistics of maintaining practice [12]. Hospital managers should focus on advocating for the continuous measurement of HHC quality, ensuring that HHC members have training before they start working in HHC, and paying attention to patients’ complaints [32].

Strengths and limitations

Data were collected using validated and reliable self-administered questionnaires. Furthermore, the randomization and adequate sample size were representative of the region. However, this study had some limitations. First, it was a cross-sectional study; therefore, it had limitations in evaluating causality and temporality. Second, this study was conducted only among Al-Ahsa physicians. Therefore, this regional focus might differ from other regions of Saudi Arabia in terms of systems and pathways. Moreover, it is a self-administered survey, which can create bias.

## Conclusions

The results of this study indicate low KAP scores among PHC physicians in Al-Ahsa, Saudi Arabia. Moreover, it highlights the need for systemic changes to improve the effectiveness of HHC delivery in Saudi Arabia. The findings of this study suggest that exposing PHC physicians to the HHC field will empower them to deliver more comprehensive, coordinated, and high-quality home-based care services.

To improve the quality and effectiveness of HHC services provided by PHC physicians, several strategic recommendations are proposed. First, it is essential to implement formal and structured training programs in HHC for family medicine residents as part of their residency curriculum. This will ensure that future physicians are adequately prepared to deliver home-based care. Second, integrating HHC content into undergraduate and postgraduate medical education can build foundational knowledge and foster a positive attitude toward home care services. Finally, establishing clear and standardized care pathways for referral to HHC services, along with improving communication mechanisms between PHC providers and HHC teams, is crucial for ensuring coordinated, efficient, and patient-centered care delivery.

## Appendices

**Table 8:** Questionnaire

Sociodemographic characteristics				
Gender	Male		Female	
Age	20-30	31-40	More than 40	
Occupation	General practitioner	Resident	Specialist	Consultant
Years of experience	Less than five years	Five to 10 years	More than five years	
I have received training in home health care	Yes		No	
I have attended lectures about home health care	Yes		No	
Did you work in home health care department before?	Yes		No	
Knowledge items				
I have adequate knowledge of home health care.	Yes/no/I don’t know			
Home health care services are limited only to the elderly.	Yes/no/I don’t know			
One of the home health care services is providing care or patients with chronic diseases.	Yes/no/I don’t know			
One of the home health care services is providing care for patients with pressure ulcers.	Yes/no/I don’t know			
One of home health care services is providing medicines, medical supplies and supplements for patients.	Yes/no/I don’t know			
Home health care provides palliative care.	Yes/no/I don’t know			
Home health care provides postpartum care.	Yes/no/I don’t know			
Home health care provides oral health.	Yes/no/I don’t know			
Home health care provides Intravenous therapy services.	Yes/no/I don’t know			
Home health care provides physiotherapy services.	Yes/no/I don’t know			
Home health care provides care for patients with urinary catheterization.	Yes/no/I don’t know			
Home health care provides care for enteral nutrition patients (patients on NGT and PEG feeding).	Yes/no/I don’t know			
Home health care provides laboratory tests.	Yes/no/I don’t know			
Home health care provides psychological and social support.	Yes/no/I don’t know			
Home health care arranges non-emergency transportation for non-ambulatory patients.	Yes/no/I don’t know			
Pressure ulcers are staged into three stages.	Yes/no/I don’t know			
FRAIL scale is used to assess risk for fall and dizziness in elderly.	Yes/no/I don’t know			
The STEADI questionnaire is used to assess depression in elderly.	Yes/no/I don’t know			
Attitude items				
I have a clear understanding of the role of home health care within my specialty.	Never/seldom/sometimes/often/always			
I am confident to in completing referral request to home health care for patients.	Never/seldom/sometimes/often/always			
I am confident in evaluating wounds and manage them accordingly.	Never/seldom/sometimes/often/always			
I am confident in performing a complete mental status examination.	Never/seldom/sometimes/often/always			
I am confident in performing a comprehensive geriatric assessment.	Never/seldom/sometimes/often/always			
Practice items				
I have referred patients to home health care.	Never/seldom/sometimes/often/always			
I have provided care to home health care patients in their homes.	Never/seldom/sometimes/often/always			
I take a detailed medical history prior to referring patients to home health care.	Never/seldom/sometimes/often/always			
I perform a thorough physical examination prior to referring patients to home health care.	Never/seldom/sometimes/often/always			
I write a detailed plan of care prior to referring patients to home health care.	Never/seldom/sometimes/often/always			
I can easily communicate with home health care providers.	Never/seldom/sometimes/often/always			
I can make adjustments to the plan of care of patients receiving home health care.	Never/seldom/sometimes/often/always			
How frequently do you refer patients to home health care services each month?	Never/seldom/sometimes/often/always			
Are there any barriers to referring patients to home health care services?	Yes/no			
Mention barriers.	Number of patients	Service not available	No experience in home health care	
Source of information regarding home health care	Media	Workshops/lecture	Experience	Self-study
